# Improvement of child art program for tsunami evacuation support 

**Published:** 2019-08

**Authors:** Moriwaki Tamaho, Yamamoto Toshiya, Homma Kenichi

**Affiliations:** ^*a*^Odawara Junior College, Japan.; ^*b*^Meiji University, Japan.

## Abstract

**Background:**

In tsunami evacuation learning for children, it is basic to give top priority to self-help, but education on evacuation support for elderly people is an issue. The contents of evacuation of elderly were introduced to "Tsunami evacuation learning art program for children", and the effect of promoting risk communication was recognized. However, when the children provided evacuation support for the elderly the current situation in which they could not evacuate within the tsunami arrival time became clear, and the measures became an issue.

This art program makes three elements important.

1. Play in the neighborhood

2. Know the neighborhood

3. Feel the emotional movement

Objective: The purpose is to improve the learning art program so that children can safely evacuate a tsunami during a disaster.

**Methods:**

A program was conducted to determine whether 46 children in Shimoda City, Shizuoka Prefecture, where tsunami disasters are expected, would help the elderly on the way to the evacuation site. The children carried the elderly to the evacuation site while being puzzled, but most people could not escape within the tsunami arrival time. We interviewed then questionnaire (40 valid respondents) and related parties before and after the implementation to conduct research. At the time of the tsunami, there were less responses to agree that it was important for me to escape, and there was a conflict over self-help and co-help.

**Results:**

Parents, "I was impressed that evacuating the elderly is wonderful, but as a parent I want the life of the child to be the top priority. We should discuss evacuation of the elderly in the area." People who experienced the tsunami say, "I was moved by the feelings of the children. The adults should be able to fulfill the feelings of the children that they want to help the elderly." It has become clear that it is the role of adults in the area to prepare for.

**Conclusions:**

Promote the intergenerational risk communication by stimulating the awareness of disaster prevention towards others, after the program for children is implemented, and the adults receive the behavior of the children and discuss "refuge for the elderly" specifically Improved to the program. In the future, we will work on the operation of the improved program.

**Keywords:**

Tsunami evacuation, Art, risk communication, Children Program, Elderly people evacuation

